# Supplementary material for: Automated counting of Drosophila imaginal disc cell nuclei
Source: Biol Open. 2024 Feb 22;13(2):bio060254. doi: 10.1242/bio.060254 (PMC10903266; doi:10.1242/bio.060254)
Supplement: Supplementary information [file biolopen-13-060254-s1.pdf]

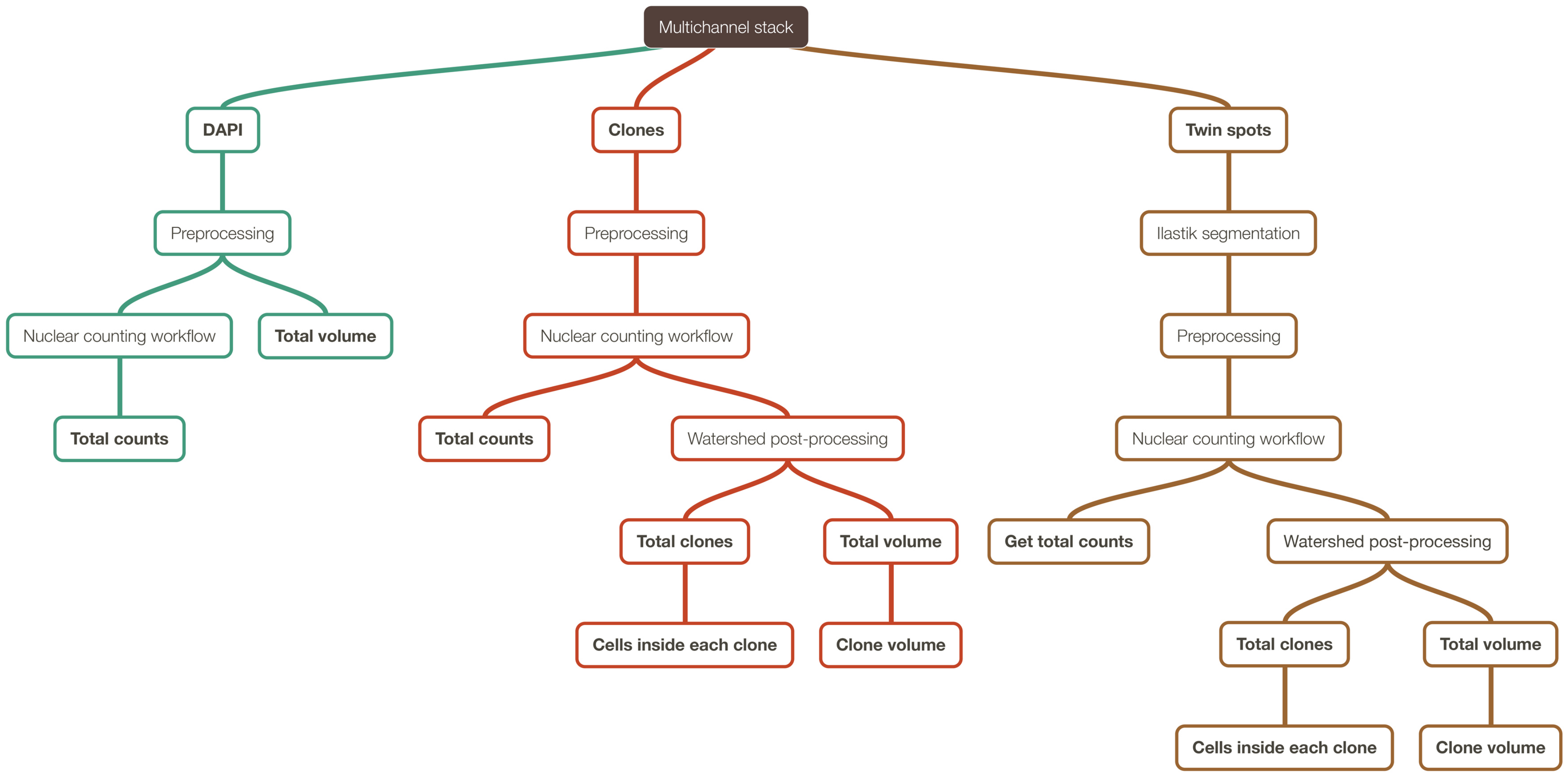

Fig. S1. Summary of the three nuclear counting workflows. Overall workflow for classification and counting of wing disc nuclei, summarizing the measurements obtained with our method.

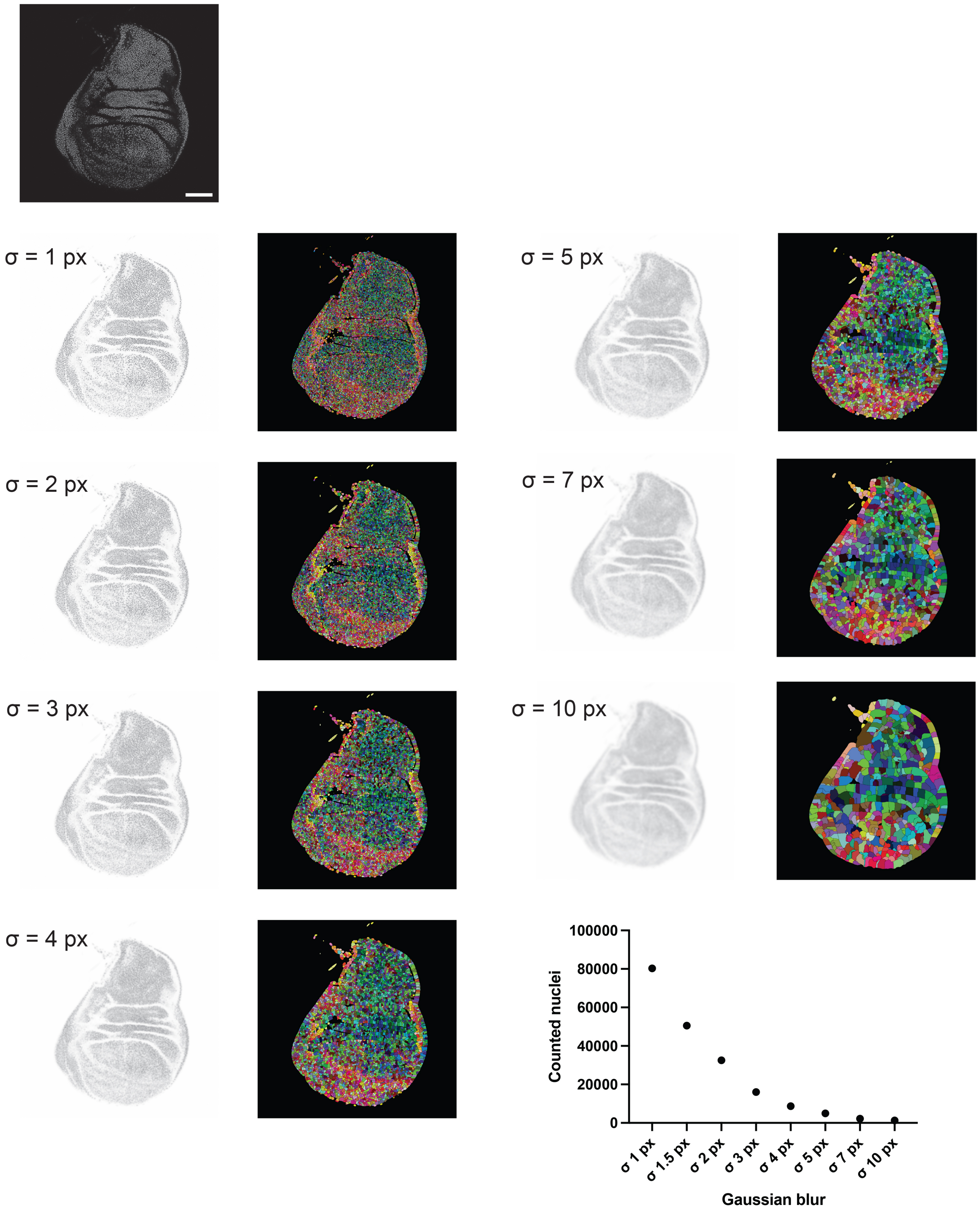

Fig. S2. Effect of gaussian blur on counting efficiency.

Images show the overall look of the disc and the resulting watershed for different sigma values of the 3D Gaussian blur on a representative DAPI-stained disc. The graph shows the counts on the example disc for the different sigma values. Scale bar: 100 µm

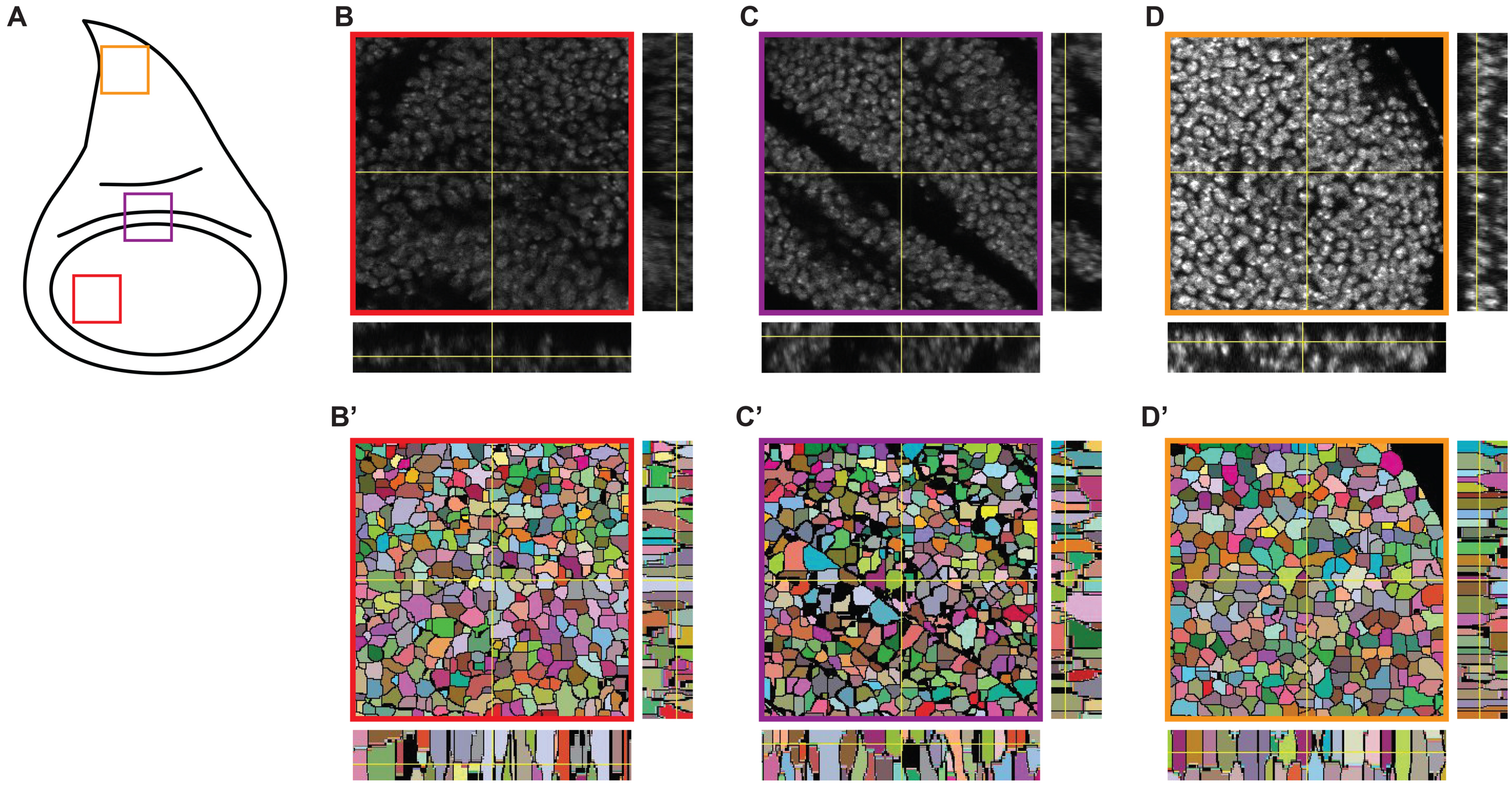

Fig. S3. Representative area used for manual versus automated counting.
A) Overview of the imaginal wing disc and the three regions chosen for manual counting, covering pouch (red), hinge (purple) and notum (orange).
B-B’) Z-slice and orthogonal views of a representative pouch region, showing the original (B) image and the segmented (B’) image resulted from the automated counts.
C-C’) Z-slice and orthogonal views of a representative hinge region, showing the original (C) image and the segmented (C’) image resulted from the automated counts.
D-D’) Z-slice and orthogonal views of a representative notum region, showing the original (D) image and the segmented (D’) image resulted from the automated counts.

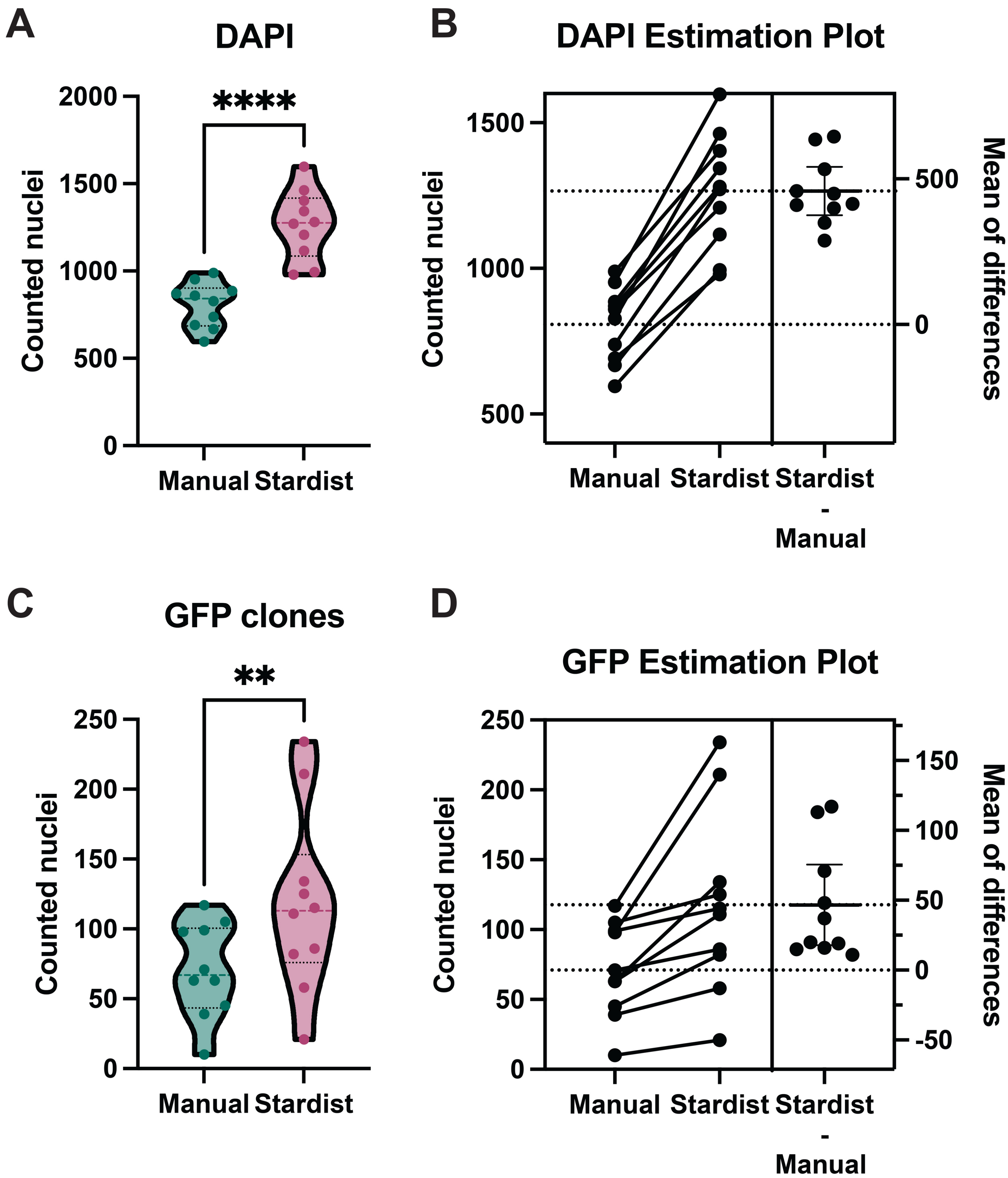

Fig. S4. Assessing the efficiency of pre-trained 3D StarDist to segment nuclei in imaginal discs.

A) Violin plot showing the differences between manually counted DAPI-stained sections against 3D StarDist. Data was analyzed using a two-tailed, paired t-test, returning a p-value < 0.0001.

B) Differences between the paired samples. Average cell counts are 807.3 manually counted nuclei versus 1265 using StarDist, with a mean ± SD difference of 458 ± 115.7 nuclei.

C) Violin plot showing the differences between manually counted GFP clones against 3D StarDist. Data was analyzed using a two-tailed, paired t-test, returning a p-value of 0.0053.

D) Differences between the paired samples. Average cell counts are 71 manually counted nuclei versus 117.7 using StarDist, with a mean ± SD difference of 46.7 ± 40.45 nuclei.

Biology Open • Supplementary information

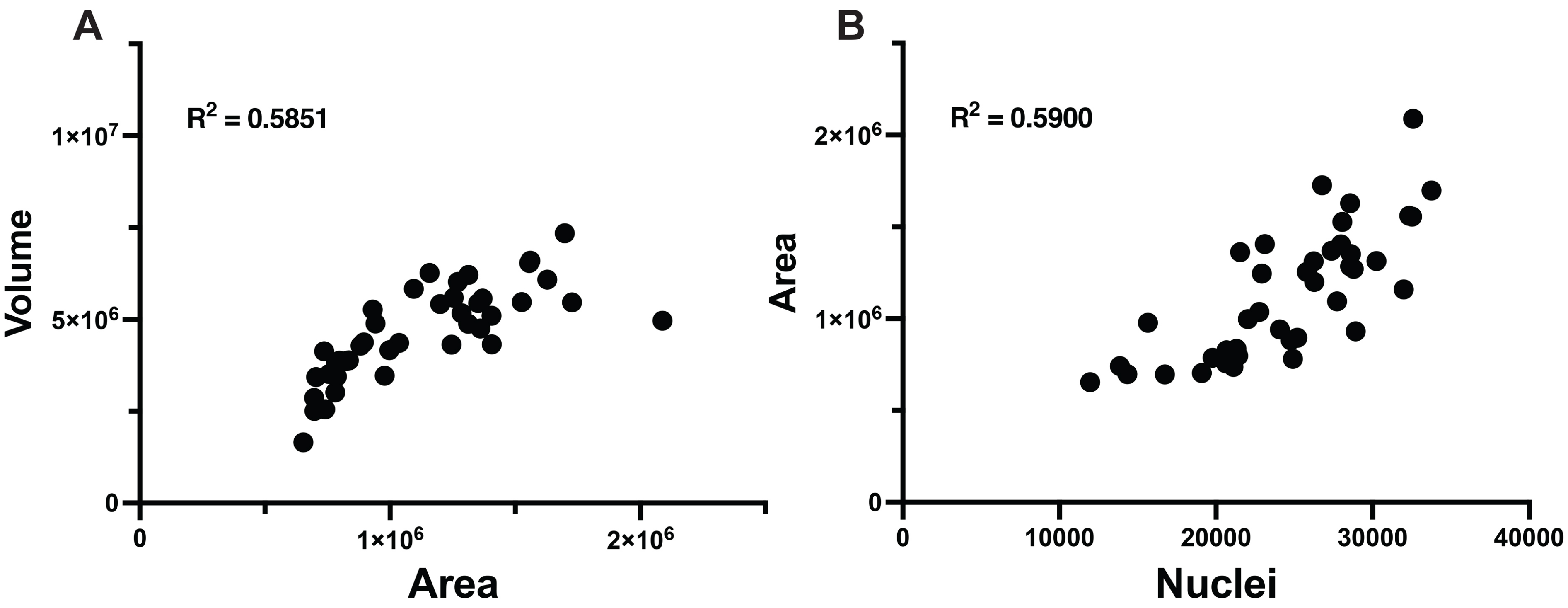

Fig. S5. Quantifying disc size using area measurements.

A) Correlation plot showing the results of measuring disc size by area vs volume. The R^2 of 0.7212 indicates that the measures don't correlate significantly.

B) Correlation between area and nuclear density. The number of nuclei does not correlate with the area the wing disc covers (R^2 of 0.5909).

Biology Open • Supplementary information

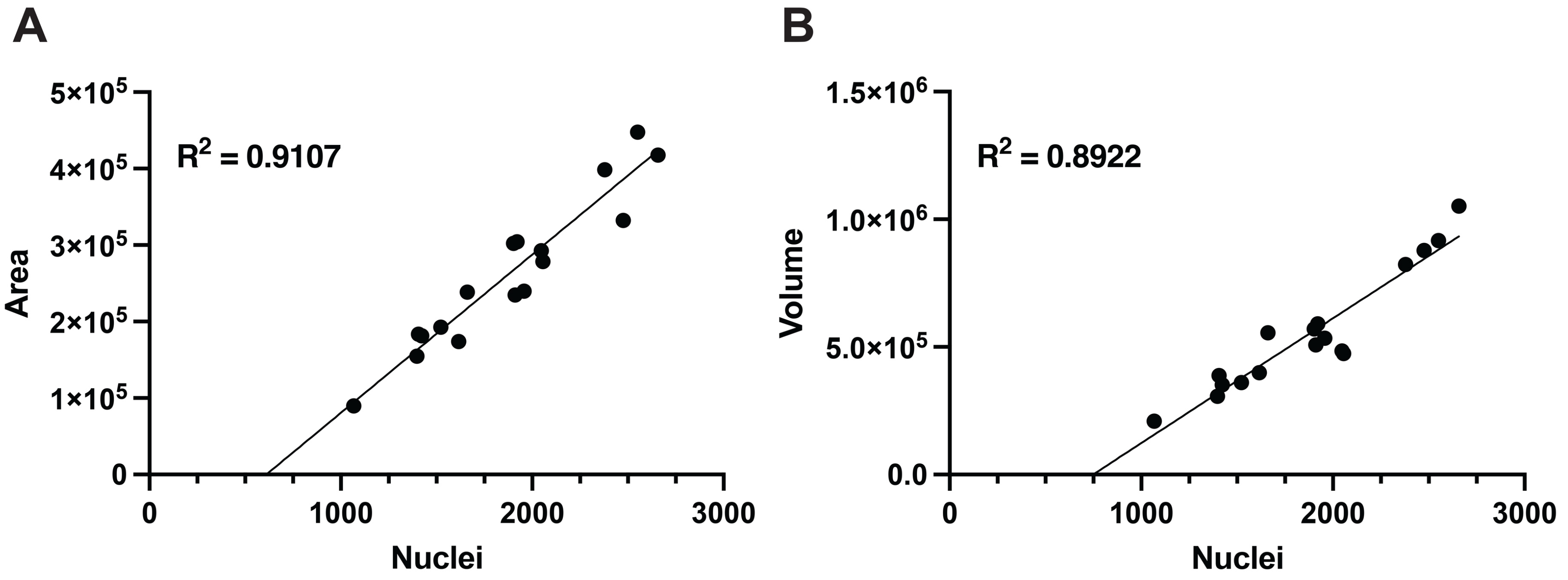

Fig. S6. Correlation between area, volume and nuclear counts in lowdensity clones.

A) Correlation between area and nuclear counts for the 17 discs in the NLS::GFP dataset used for clone counts (R^2 of 0.9107).

B) Correlation between volume and nuclear counts for the same dataset (R^2 of 0.8922).

Biology Open • Supplementary information

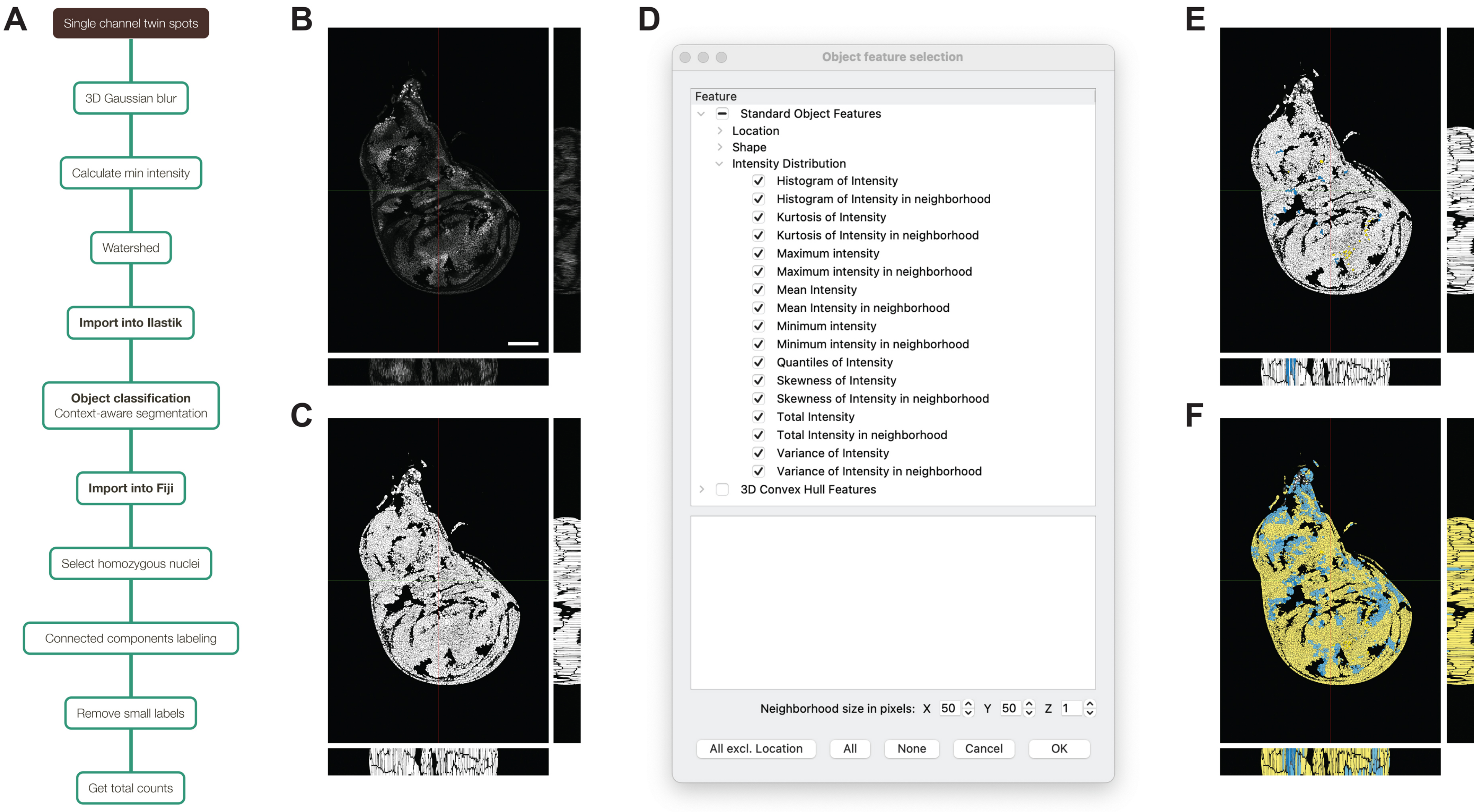

Fig. S7. Twin-spot classification overview.

A) Schematic of the segmentation of homozygous twin-spots using Fiji and Ilastik's Object classification.
B) Raw input image passed on to Ilastik for the clone classification.
C) Segmentation image used by Ilastik to classify each nucleus.
D) Computation parameters used to classify each nucleus as heterozygous or homozygous according to its intensity and the surrounding 50x50 pixels.
E) Training of the object classification algorithm, showing a few nuclei manually classified as heterozygous (yellow) or homozygous (blue).
F) Prediction for every nRFP nucleus as heterozygous (yellow) or homozygous (blue) in the wing disc based on the trained algorithm. All wing disc images show the central slice of the Z stack, with the orthogonal views corresponding to the sections highlighted in green and red. Scale bar: 100 µm

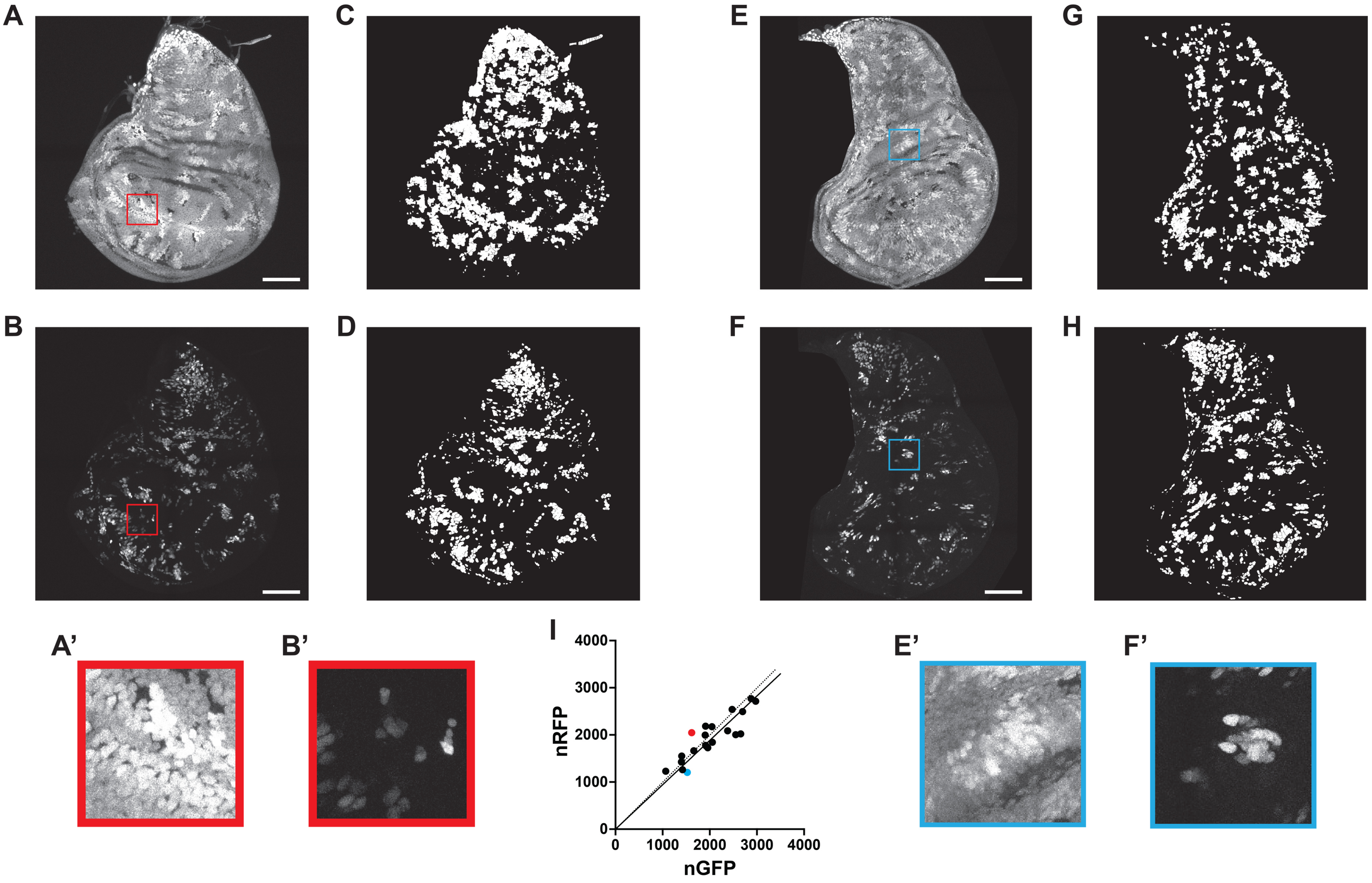

Fig. S8. Differential expression of tub-nRFP and UAS-nGFP in twin spot clones.

A-D) Representative disc in which GFP nuclei are more numerous than RFP nuclei.

E-H) Representative disc in which GFP nuclei are less abundant than RFP nuclei.

A, E) nRFP twin-spot nuclei.

B, F) Homozygous nRFP twin-spot nuclei classified by Fiji and Ilastik as summarized in the methods.

C, G) GFP nuclei.

D, H) Segmentation of GFP nuclei.

I) Correlation plot comparing nRFP and nGFP in 22 discs. The representative disc in (A) is indicated by the red dot and in (E) by the blue dot. Scale bar: 100 μm

Biology Open • Supplementary information
